# Gut microbial methionine impacts circadian clock gene expression and reactive oxygen species level in host gastrointestinal tract

**DOI:** 10.1093/procel/pwac021

**Published:** 2022-07-15

**Authors:** Xiaolin Liu, Yue Ma, Ying Yu, Wenhui Zhang, Jingjing Shi, Xuan Zhang, Min Dai, Yuhan Wang, Hao Zhang, Jiahe Zhang, Jianghua Shen, Faming Zhang, Moshi Song, Jun Wang

**Affiliations:** CAS Key Laboratory of Pathogenic Microbiology and Immunology, Institute of Microbiology, Chinese Academy of Sciences, Beijing 100101, China; University of Chinese Academy of Sciences, Beijing 100049, China; CAS Key Laboratory of Pathogenic Microbiology and Immunology, Institute of Microbiology, Chinese Academy of Sciences, Beijing 100101, China; University of Chinese Academy of Sciences, Beijing 100049, China; CAS Key Laboratory of Pathogenic Microbiology and Immunology, Institute of Microbiology, Chinese Academy of Sciences, Beijing 100101, China; University of Chinese Academy of Sciences, Beijing 100049, China; CAS Key Laboratory of Pathogenic Microbiology and Immunology, Institute of Microbiology, Chinese Academy of Sciences, Beijing 100101, China; University of Chinese Academy of Sciences, Beijing 100049, China; CAS Key Laboratory of Pathogenic Microbiology and Immunology, Institute of Microbiology, Chinese Academy of Sciences, Beijing 100101, China; CAS Key Laboratory of Pathogenic Microbiology and Immunology, Institute of Microbiology, Chinese Academy of Sciences, Beijing 100101, China; Medical Center for Digestive Diseases, the Second Affiliated Hospital of Nanjing Medical University, Nanjing 210011, China; University of Chinese Academy of Sciences, Beijing 100049, China; State Key Laboratory of Membrane Biology, Institute of Zoology, Chinese Academy of Sciences, Beijing, 100101, China; Institute for Stem cell and Regeneration, Chinese Academy of Sciences, Beijing, 100101, China; Beijing Institute for Stem Cell and Regenerative Medicine, Beijing 100101, China; University of Chinese Academy of Sciences, Beijing 100049, China; State Key Laboratory of Membrane Biology, Institute of Zoology, Chinese Academy of Sciences, Beijing, 100101, China; Institute for Stem cell and Regeneration, Chinese Academy of Sciences, Beijing, 100101, China; Beijing Institute for Stem Cell and Regenerative Medicine, Beijing 100101, China; University of Chinese Academy of Sciences, Beijing 100049, China; State Key Laboratory of Membrane Biology, Institute of Zoology, Chinese Academy of Sciences, Beijing, 100101, China; Institute for Stem cell and Regeneration, Chinese Academy of Sciences, Beijing, 100101, China; Beijing Institute for Stem Cell and Regenerative Medicine, Beijing 100101, China; University of Chinese Academy of Sciences, Beijing 100049, China; State Key Laboratory of Membrane Biology, Institute of Zoology, Chinese Academy of Sciences, Beijing, 100101, China; Institute for Stem cell and Regeneration, Chinese Academy of Sciences, Beijing, 100101, China; Beijing Institute for Stem Cell and Regenerative Medicine, Beijing 100101, China; Medical Center for Digestive Diseases, the Second Affiliated Hospital of Nanjing Medical University, Nanjing 210011, China; Key Lab of Holistic Integrative Enterology, Nanjing Medical University, Nanjing 210011, China; University of Chinese Academy of Sciences, Beijing 100049, China; State Key Laboratory of Membrane Biology, Institute of Zoology, Chinese Academy of Sciences, Beijing, 100101, China; Institute for Stem cell and Regeneration, Chinese Academy of Sciences, Beijing, 100101, China; Beijing Institute for Stem Cell and Regenerative Medicine, Beijing 100101, China; CAS Key Laboratory of Pathogenic Microbiology and Immunology, Institute of Microbiology, Chinese Academy of Sciences, Beijing 100101, China; University of Chinese Academy of Sciences, Beijing 100049, China

**Dear Editor,**

Circadian rhythms are the periodic turnover of biological behaviors and physiological functions of many organisms (Allada and Bass, 2021). In mammals, circadian rhythms are maintained by the clock system, involving central clock in suprachiasmatic nucleus and peripheral clock in organs like intestine; and at cellular level a cell-autonomous transcriptional and translational feedback loop involving clock genes like *BMAL1* (brain and muscle ARNT-Like 1, also known as *ARNTL*), *CLOCK* (clock circadian regulator), *PER1*/*2*/*3* (period circadian regulator 1/2/3) and *CRY1*/*2* (cryptochrome circadian regulator 1/2) (Allada and Bass, 2021). Accumulating evidence indicates that circadian rhythm disorders affect host neurological, metabolic, and immunological homeostasis, consequently contributing to a list of neurodegenerative diseases, metabolic diseases, and cancers (Holth Jerrah et al., 2019; Allada and Bass, 2021).

Gut microbes also have their own circadian rhythm and recent advances in mice reveal that the composition, function, and biogeography of gut microbiota undergo circadian oscillations, which are controlled by the timing of food intake and diet compositions and have the potential to influence the gastrointestinal tract and other organs by bacterial antigens and metabolites (Thaiss et al., 2014, 2016). So far, such studies have been largely limited in mice and only few investigated rhythms in the human gut microbiota, such as our recent *in situ* survey (Liu et al., 2021), which identified many bacterial taxa and metabolic pathways with circadian oscillations. However, whether and how clock of gut microbiome affects host circadian rhythms as well as health remain to be explored. Therefore, we here firstly identified the important functions with same circadian pattern in both human (aforementioned *in situ* survey) and mouse gut microbiome, and then further investigated the possible effects of metabolite involved in the function on the host.

To investigate the shared rhythm-related metabolic features in mouse and human gut microbiome, we firstly collected feces from 10 mice at 10:00 and 22:00 and performed metatranscriptome sequencing and metabolome profiling. Comparing the transcriptional profiles, we found that mouse gut microbiota composition and function showed significant day-night differences (Fig. 1A), and the richness and evenness of gut microbiota observed at night have a trend of relatively higher levels (Fig. S1A and S1B). In addition, we identified the marker species with significant day-night differences was *Helicobacter bilis* and in terms of metabolic pathways, *S*-adenosyl-l-methionine cycle I, methylerythritol phosphate pathway I, l-lysine biosynthesis III, and seleno-amino acid biosynthesis showed significant differences between day and night (Fig. 1B). Among them, two metabolic pathways shared between human and mice were identified, and *S*-adenosyl-l-methionine cycle I was the only showing a same oscillation pattern, namely increased metabolic activity during wake period of both human (day) and nocturnal mice (night), and reversely in the sleep period (Fig. 1C). Then, the metabolomic profiling focusing on day-night alterations in composition of fecal metabolites (Fig. 1D) found a total of 24 fecal metabolites significantly accumulated during the night (wake phase), including l-methionine, Racemethionine, and Methionine sulfoxide. Additionally, eight metabolites showed opposite pattern, such as *N*, *N*-Dimethylsphingosine, *N*-Methylnicotinamide, and Sphingosine (Figs. 1E and S1C). Further enrichment analysis identified 14 metabolic pathways and 2 were markedly affected by day-night cycles, with methionine metabolism ranked as the most significant pathway (Fig. S1D). Thus, the methionine and related metabolic pathways here were found to be of significant circadian pattern and were investigated further.

**Figure 1.**
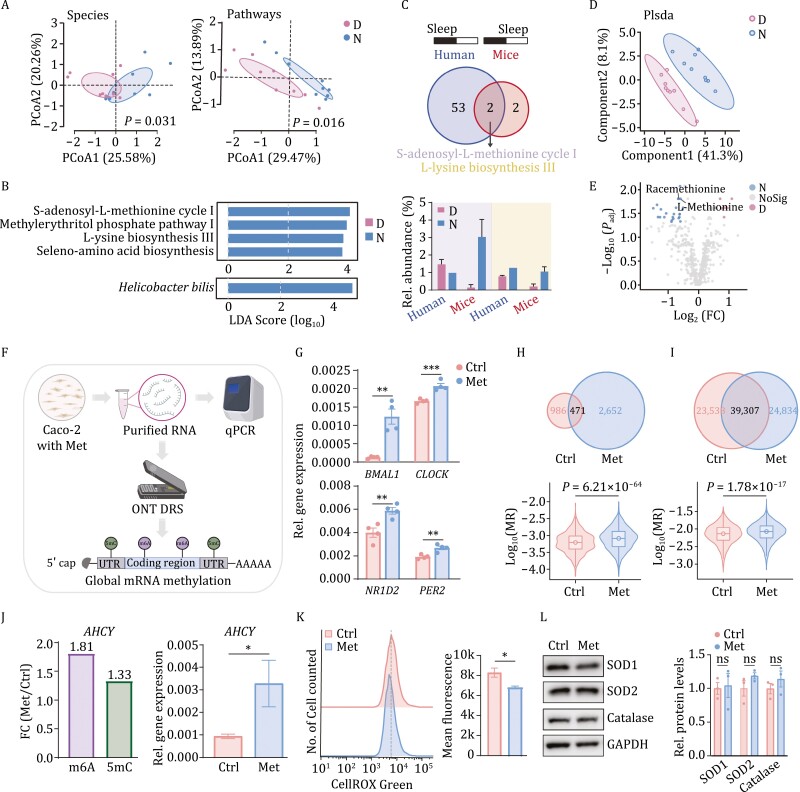
Key gut microbial metabolite with circadian oscillation identifying and its potential effects on Caco-2 cell. (A) Significant day-night differences were observed in both composition (left panel) and function (right panel). D: daytime; N: nighttime. The significances of how two group can be distinguished were calculated using envfit function in vegan package in R. (B) Specie (bottom panel) and pathways (top panel) showed significant day-night difference (LDA score [log_10_] < −2 or > 2; *P*_adj_ < 0.05). (C) The intersection of metabolic pathways with day-night difference of mouse gut microbiome in this study and that of human gut microbiota in our previous *in situ* study (Liu et al., 2021) identified two shared metabolic pathways (top panel) and *S*-adenosyl-l-methionine (SAM) cycle I showed a same activity oscillation pattern, namely increased metabolic activity during wake phase of both human (day) and nocturnal mice (night), and reversely in the sleep phase (bottom panel). Different colors indicate the two shared pathways, the purple one representing *S*-adenosyl-l-methionine cycle I and the yellow one referring to l-lysine biosynthesis III. The “sleep” in the top panel indicates sleep phase of human or mouse. (D) Metabolomic profiling showed alterations between day and night fecal samples. (E) Twenty-four metabolites with night preference and eight accumulating during the daytime were identified with fold change (FC) < 0.5 or > 2; *P*_adj_ < 0.05. (F) The simple workflow of relative expression of for core clock genes in methionine-treated Caco-2 cells with Quantitative Real-time PCR (qPCR) and the cellular mRNA methylation level detecting with Oxford Nanopore technology direct RNA sequencing (ONT DRS). (G) The relative gene expression of four circadian clock genes, namely *BMAL1*, *CLOCK*, *NR1D2*, *PER2* were detected in Caco-2 cells after vehicle (10% PBS) or 100 μmol/L methionine (Met group) treated for 24 h. (H) More m6A modified sites (top panel) and higher overall levels of m6A modification rate (MR, bottom panel) were detected in Caco-2 cells under 100 μmol/L methionine treatment compared to the Ctrl (PBS-treated) group. (I) More 5mC modified sites (top panel) and higher overall levels of 5mC MR (bottom panel) were detected in Caco-2 cells under 100 μmol/L methionine treatment compared to the Ctrl group. (J) The increased mRNA methylation levels (left panel) and relative gene expression level (right panel) of *AHCY* were detected with fold change analysis and qPCR. (K) The cellular reactive oxygen species (ROS) levels significantly decreased by methionine treatment in Caco-2 cells with CellROX Green flow cytometry analysis. (L) Western blot analyses showed that methionine has no significant effect on all the three antioxidant enzymes, superoxide dismutase1 (SOD1), superoxide dismutase2, (SOD2) and catalase in Caco-2 cells. The “n.s.” means no significant different in relative protein levels of all the three antioxidant enzymes. Values are presented as means ± s.e.m. Statistical significance was declared at 0.05. *P < 0.05, **P < 0.01, ***P < 0.005, ****P < 0.001.

To investigate the potential effects of methionine on host circadian rhythms, we firstly treated Caco-2 cells, an often-used model of intestinal barrier function, with 100 μmol/L methionine for 24 h, and checked the cellular expression of four core clock genes, namely, *BMAL1*, *CLOCK*, *NR1D2* (nuclear receptor subfamily 1 group D member 2) and *PER2* (Fig. 1F). The mRNA clues showed significant increases in expression of these genes after methionine treatment (Fig. 1G). Given that the methionine can serve as a dietary methyl donor as the precursor of *S*-adenosyl-l-methionine (SAM) (Li et al., 2020), we utilized the state-of-the-art Oxford Nanopore technology (ONT) to perform direct RNA sequencing (DRS) of the Caco-2 cells. Then, mRNA N6-methyladenosine (m6A) and 5-methylcytosine (5mC) modifications were detected (Fig. 1F), and we identified 1,572 and 3,302 significant m6A modified sites in vehicle- and methionine-treated Caco-2 cells, respectively (*P* < 0.001, DMR > 0.5; DMR refers to differential modification rates). Among these, only 471 were shared by cells across groups and a systematic increase in modified sites after methionine treatment (Fig. 1H). Also, 69,678 and 70,317 5mC modified sites were identified in control and methionine-treated groups and only 39,307 sites were shared (Fig. 1I). Then, these modified sites were used to calculate m6A and 5mC modification rate (MR) of transcripts, and the methionine-treated cells were shown to have markedly higher MR (Fig. 1H and 1I).

The transcripts were then aligned to gene coordinates and MRs of genes were calculated and 900 and 2,606 genes with increased m6A and 5mC modification levels in cells treated by methionine were identified, respectively (fold change (FC) > 1). Then, many rhythm-related genes with increased m6A (29/900) and 5mC (77/2,606) levels were identified by querying in GO databases (Fig. S2). More importantly, we observed increased m6A and 5mC level of *AHCY* (adenosylhomocysteinase) gene (FC of m6A = 1.81, FC of 5mC = 1.33), protein product of which was recently reported to be essential for cyclical H3K4 trimethylation and recruitment of BMAL1 to chromatin and promoting subsequent circadian transcriptional activity (Greco Carolina et al., 2020). Likewise, the expression of *AHCY* was also observed to be up-regulated in methionine-treated cells (Fig. 1J). Altogether, methionine could regulate clock gene expression of human intestinal cells and increase overall cellular methylation, casting effect on overall rhythm-related genes, in which the increased mRNA methylation level and accumulated mRNA of *AHCY* gene serve as a possible link.

Another important reported consequence of circadian rhythm disruption (sleep deprivation; SD) is the accumulation of intestinal reactive oxygen species (ROS), a major detrimental substance causing cellular oxidative damaging and necrosis (Vaccaro et al., 2020). Additional evidence also points to gut microbiota potentially modulating SD-induced inflammatory response and cognitive impairment (Wang et al., 2021). Given that methionine is a reductive amino acid (Li et al., 2020), we investigated methionine’s additional effect on reducing ROS. First, a significant lower ROS level was detected in methionine-treated Caco-2 cells (Fig. 1K). However, for three antioxidant enzymes, including superoxide dismutase 1 (SOD1), superoxide dismutase 1 (SOD2), and catalase, methionine did not significantly increase their intracellular levels (Fig. 1L). thus, methionine potentially decreases cellular ROS by serving as a direct antioxidant.

We then tested the antioxidant activity of methionine in a sleep-deprived (SD) mouse model. We employed four groups of mice, one control group with normal diet and sleep-wake cycle (Normal), and three SD groups with dietary methionine manipulation (0%, 0.86%, 1.36%) subjected to 3 days of SD in a restriction chamber with a constantly and gently rotating bar (Met-free SD, Normal SD, and Met-rich SD) (See Materials and Methods) (Fig. 2A). Firstly, significant loss in body weights was observed despite the elevated dietary intake, especially for the Met-free SD group (Fig. 2B). Moreover, the serum corticosterone, a known regulator of circadian physiology, were increased significantly in the SD groups and the methionine supplementation reduced levels of circulating corticosterone, indicating that methionine has the potential to mitigate SD-induced increase of corticosterone (Fig. 2D).

**Figure 2.**
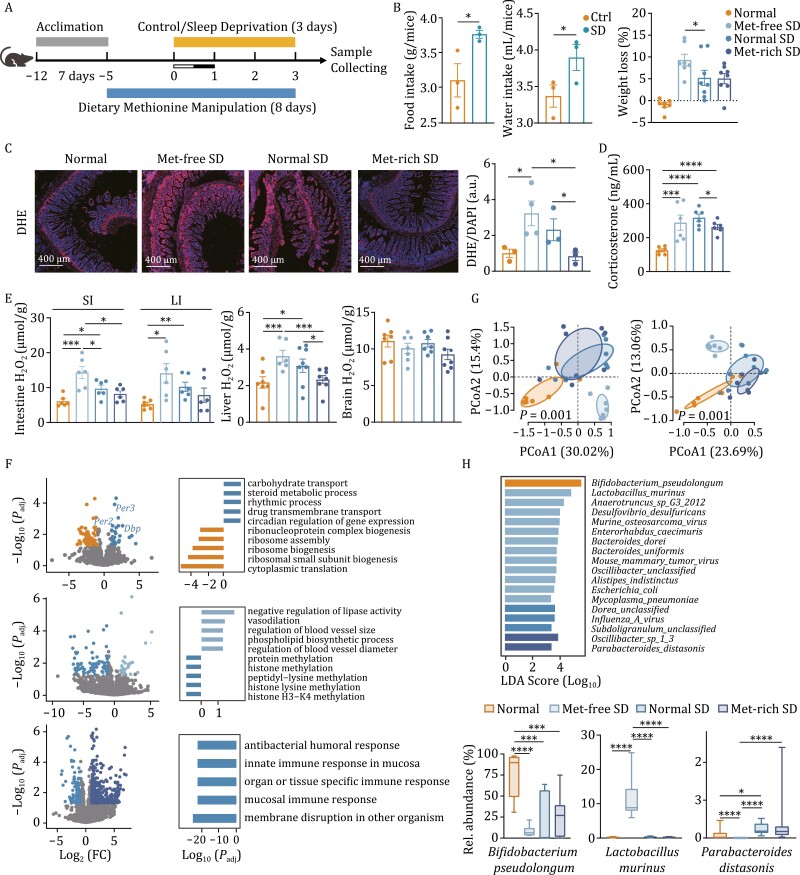
Protective effects of methionine on a sleep-deprived mouse model. (A) Schematic diagram of the mice sleep deprivation (SD) study. (B) Food and water intake of three SD groups of mice before and during the SD period (left panel) and the weight loss of four groups during the SD period (right penal). SD had significantly increased diet intake; however, it significantly decreased mice body weight, especially for the Met-free group (0% methionine in diet). (C) DHE staining of mice small intestine. Higher ROS levels were seen in the small intestines after 3 days of SD and methionine deprivation/supplementation aggravated/relieved the ROS accumulation. The red channel refers to DHE and blue channel refers to DAPI. The right panel is the quantification of ROS levels. (D) Serum corticosterone levels of mice. Circulating corticosterone accumulated after SD and supplementation of methionine significantly reduced serum corticosterone levels. (E) H_2_O_2_ levels of intestines (Left panel), liver (middle panel), and brain (right panel) from SD and non-SD mice. SI means small intestine and LI presents large intestine. (F) RNA-seq analyses of small intestinal tissue of SD and non-SD mice. Volcano plots displaying differentially expressed genes in comparisons of Normal SD and Normal (top panel), Met-free SD and Normal SD (middle panel), Met-rich SD and Normal SD (bottom panel), respectively. Different colors represent different groups and the colored dots represent differentially expressed genes with log_2_ (FC) > 1, *P*_adj_ < 0.05. The bar plots showing the most enriched biological functions (top 5) in GO enrichment analysis of differentially expressed gene in comparisons of Normal SD and Normal (top panel), Met-free SD and Normal SD (meddle panel), Met-rich SD and Normal SD (bottom panel), respectively. The significance declared at *P* < 0.05 and *P*_adj_ < 0.20. (G) Principal coordinate analysis (PCoA) showing significant different in composition (left panel) and function (right panel) of SD and non-SD mouse gut microbiota. (H) The differential species across groups were identified with Linear discriminant analysis (LDA) effect size (LEfSe) analysis (top panel) with LDA score (log_10_) > 2 and *P*_adj_ < 0.05 and the distributions of relative abundances of three important dominant species in the Normal, Met-free SD and Met-rich SD groups, respectively. Values are presented as means ± s.e.m. Statistical significance was declared at 0.05. *P < 0.05, **P < 0.01, ***P < 0.005, ****P < 0.001.

Then, intestinal ROS were analyzed using dihydroethidium (DHE) staining, in which we found that, the SD mice (Normal SD vs. Normal group) showed significantly increased intestinal ROS level, in line with previous report (Vaccaro et al., 2020). Furthermore, for SD mice, methionine level had effect on intestinal ROS, as significantly reduced ROS in the Met-rich group and increased ROS levels in mice fed with Met-free chow (Fig. 2C). Also, we detected H_2_O_2_ level of intestine, liver and brain and observed a similar effect pattern of dietary methionine in the examined tissues except for brain as that of ROS in the small intestine (Fig. 2E). Thus, methionine can function as a powerful antioxidant compound not only *in vitro* but also *in vivo*.

Given that SD has several other reported deleterious effects (Wang et al., 2021), we then investigated the impact of methionine on these effects. We here performed RNA-seq in murine small intestines, livers, and blood cells and transcriptomic analysis revealed that, in intestine, SD (Normal SD vs. Normal) up-regulated the expression of some circadian genes, including *Per2*, *Per3*, and *Dbp* (D-box binding PAR bZIP transcription factor). Enrichment analysis demonstrated that small intestines of SD mice had multiple biological processes related to circadian rhythms up-regulated (Fig. 2F). Moreover, dietary methionine restriction led to higher expression of genes related to vasodilation in SD mice and, conversely, down-regulation of methylation-related genes. Furthermore, in circulatory blood cells, the Met-free SD group (with the highest ROS levels in intestines) showed significant inflammatory signals like cellular response to granulocyte macrophage, chemokine biosynthetic process, and cytokine biosynthetic process (Fig. S4). Conversely, methionine supplementation down-regulated genes related to functions like mucosal immune response and innate immune response in these tissues, suggesting that methionine potentially reduce SD-induced systemic inflammation and its neutralizing effect on ROS might be relevant (Figs. 2F, S3 and S4).

Moreover, metagenomic analysis indicated the SD-induced gut microbiota dysbiosis, in concert with previous report (Wang et al., 2021), with composition and function significantly altered and some beneficial taxa, like abundance of *Bifidobacterium pseudolongum* decreasing (log_10_ (LDA) > 2, *P*_adj_ < 0.05) and deleterious microbes, such as *Influenza A virus* and *Dorea spp* increasing. Moreover, potentially harmful microbes, like *Lactobacillus murinus*, a species which has been reported to related to intestinal dysbiosis and biotin deficiency (Hayashi et al., 2017) significantly enriched in the methionine deprived group (Met-free SD), together with several known pathogens including Murine osteosarcoma virus, Mouse mammary tumor virus, and *Mycoplasma pneumoniae* (Fig. 2G and 2H). However, these microbes were reduced with methionine supplementation, and the relative abundance of beneficial microbe like *Parabacteroides distasonis*, which was reported to improve obesity and metabolic dysfunctions (Wang et al., 2019), significantly increased, together with *Oscillibacter sp.1_3* (Fig. 2H). Meanwhile, metabolic activity of the gut microbiota also showed significant alterations across the groups (Fig. S5). Overall, methionine has additional potential to protect or restore gut microbiome from SD-induced dysbiosis.

To summarize, we pinpointed one key gut microbial metabolite, methionine, from overlapped metabolic pathways and metabolites with diurnal patterns in mice and human. Furthermore, in this study, methionine metabolism is the microbial function most affected by the diurnal cycles in mice; however, it has been reported to become arrhythmic in T2D patients and mouse model (also have a relatively lower circulating Methionine level) (Reitmeier et al., 2020); overall, these evidences support the important of methionine, and its diurnal patterns in metabolic homeostasis. We firstly established that methionine supplementation significantly increased the expression of circadian genes in Caco-2 cells. Mechanistically, we measured mRNA methylation and found that methionine supplementation indeed increased the overall methylation levels, and that of one particular gene *AHCY* that is crucial for BMAL1 recruitment and consequent circadian transcription of genes (Greco Carolina et al., 2020). Consistently, it has been shown that 3-deazaadenosine, an ACHY inhibitor shortens the circadian period of clock genes by affecting the intracellular RNA methylation (Fustin et al., 2013). Then, we tested the *in vivo* potential of methionine in a SD mouse model by dietary methionine manipulation. Although some evidence indicates that dietary methionine supplementation has various effects on the host, such as benefits like improving hepatic steatosis and insulin resistance, as well as disadvantages like increasing cholesterol levels (Navik et al., 2021). Here, we reveal that it has the pleiotropic potential to neutralize SD-induced ROS and alleviate inflammation and gut microbiota dysbiosis associated with SD. Moreover, Methionine, after being oxidized to methionine sulfoxide, can be recycled and reduced again, making it a regenerable agent for ROS reduction (Lee and Gladyshev, 2011), thus it has high potentials of pharmaceutical applications in treating common sleep deprivation and consequent adverse effects.

## Supplementary Material

pwac021_suppl_Supplementary_Material

## Footnotes

This work is supported by the National Key Research and Development Program of China (2018YFC2000500), the National Natural Science Foundation of China (91857101), the Strategic Priority Research Program of the Chinese Academy of Sciences (XDB29020000), the National Natural Science Foundation of China (81922027, 81870228, and 81921006), and Beijing Natural Science Foundation (JQ20031) and the State Key Laboratory of Membrane Biology.

The authors declare that they have no conflict of interest or financial conflicts to disclose.

All institutional and national guidelines for the care and use of laboratory animals were followed.

The metatranscriptomic and metagenomic sequencing data of mouse feces and the RNA-seq data of mouse small intestines, livers and blood cells have been deposited at the National Microbiology Data Center (NMDC10018126) and are publicly available as of the date of publication. Further information and requests for other datasets in the study are available from the Lead Contact, Jun Wang (junwang@im.ac.cn) on reasonable request.

J.W., M.S. and F.Z. conceived this study and supervised all the experiments and the manuscript. X.L. designed and performed functional experiments and analyzed and interpreted data. Y.M. and Y.Y. provided assistance with data analysis and experimental design, respectively. J.W. and X.L. wrote the manuscript. All authors read and approved the final manuscript.
