# Supplementary material for: Gut microbial methionine impacts circadian clock gene expression and reactive oxygen species level in host gastrointestinal tract
Source: Protein Cell. 2022 Jul 15;14(4):309–13. doi: 10.1093/procel/pwac021 (PMC10120970; doi:10.1093/procel/pwac021)
Supplement: pwac021_suppl_Supplementary_Material [file pwac021_suppl_supplementary_material.docx]

**Supplementary Materials**

**Title： Gut microbial methionine impacts circadian clock gene expression and reactive oxygen species level in host gastrointestinal tract**

**Running Title：** Methionine affects intestinal rhythms and oxidative stress

**Keywords:** Gut microbiota; circadian clock gene; methionine; mRNA methylation; reactive oxygen species

Xiaolin Liu^1,2^, Yue Ma^1,2^, Ying Yu^1,2^, Wenhui Zhang^1,2^, Jingjing Shi^1^, Xuan Zhang^1^, Min Dai^3^, Yuhan Wang^2,4,5,6^, Hao Zhang^2,4,5,6^, Jiahe Zhang^2,4,5,6^, Jianghua Shen^2,4,5,6^, Faming Zhang^3,7*^, Moshi Song^2,4,5,6*^, and Jun Wang^1,2,8*^

^*^Correspondence: [junwang@im.ac.cn](mailto:junwang@im.ac.cn) (J. Wang), [songmoshi@ioz.ac.cn](mailto:songmoshi@ioz.ac.cn) (M. Song), [fzhang@njmu.edu.cn](mailto:fzhang@njmu.edu.cn) (F. Zhang)

Materials and Methods, Supplemental Reference, Fig. S1–S5, and Tab. S1

**Materials and Methods**

**Mice**

Two-month-old male C57BL/6J mice were purchased from Beijing HFK Bioscience and housed in ventilated cages under strict SPF conditions in our animal facility. All mice were maintained at constant temperature (23° ± 0.5 °C) with moderate humidity (50 ± 5%) and a 12-h light/dark cycle (lights on at 07:00 h; off at 19:00h). Mice were fed ad libitum and had unlimited access to water. All housing protocols and animal experiments were approved by the Ethics Committee of the Institute of Microbiology, Chinese Academy of Sciences (permit SQIMCAS2021005).

**Cellular models**

Caco-2 cells were cultured in Eagle's Minimum Essential Medium (EMEM; ATCC; 30-2003) containing 20% Fetal Bovine Serum (FBS; GIBCO; 10099-141C) and 1% Penicillin-Streptomycin (GIBCO; 1514022). Cells were pre-treated and synchronized with 200nM dexamethasone for 1 hour (DEX; Sigma-Aldrich; D1756), followed by L-Methionine (Sigma-Aldrich; M9625) and phosphate-buffered saline (PBS; Solarbio; P1020). All cells were cultured at 37 °C with 5% CO2.

**Mice feces sampling and metatranscriptomic and metagenomic sequencing**

The day-night fecal samples of mice were freshly collected from health mice with normal sleep-wake cycle and free access to chow and water at 10:00 and 22:00 in a same day. The total of 20 fecal samples collected were snap-frozen and stored at -80℃ before analyses. The total RNA of these fecal samples was extracted and separated using a RNeasy PowerMicrobiome Kit (Qiagen, Germany). Then, rRNA in the separated total RNAs was depleted with a RiboMinu Transcriptome Isolation Kit (Thermo Fisher Scientific, US), which utilizes rRNA-complementary probes to bind and remove rRNA. The extracted RNA was sequenced on the Illumina NovaSeq PE150 platform. The feces of 30 mice with dietary methionine manipulation in the sleep deprivation experiment were also sampled at 10:00 and were frozen immediately after collection, followed by stored at -80℃ before analyses. Then, these samples were processed to extract the total gut microbiota genomic DNA using the QIAamp PowerFecal DNA Kit (Qiagen, Germany) referring to the the manufacturer’s instructions. Then, the extracted DNA was sequenced on the Illumina NovaSeq PE150 platform as well.

**Taxonomic and functional profiling**

The raw metatranscriptomic and metagenomic data were subjected to filter and remove the reads with low quality or reads mapped to host genome by KneadData (v0.7.2), calling software Trimmomatic 0.36 (Bolger et al., 2014). A total of 70,511,928 and 46,351,244 million reads then were sliced out randomly form qualified metatranscriptomic and metagenomic data of each sample for the same sequencing depth. Next, taxonomic and functional profiling were performed utilizing MetaPhlAn2 (v2.0) and HUMAnN2 (v0.11.2) as reported before (Segata et al., 2012; Franzosa et al., 2018). A total of 21 species and 145 metabolic pathways were identified in mice day-night feces; 59 bacterial species and 303 pathways were found in the mice feces in sleep deprivation (SD) experiment. Among these, the most abundant species and pathways were selected in R 3.6.3. Then, alpha diversity was assessed by Chao1 Index and Shannon evenness, and beta diversity was calculated by Bray–Curtis dissimilarity metric and reported by principal coordinate analysis in the R package vegan (v2.5-6). Next, the species and pathways showing day-night difference or functioning as important biomarker in SD mice were selected in LDA Effect Size analysis, with LDA more than 2 and Pvalue less than 0.05 (Segata et al., 2011).

**Metabolomic analysis**

Fecal samples were extracted with solution (acetonitrile: methanol: water = 2: 2: 1, with isotopically-labelled internal standard) and the supernatant was used for analysis. The quality control (QC) sample was prepared by mixing an equal aliquot of the supernatants from all of the samples. LC-MS/MS analyses were performed using an UHPLC system (Vanquish, Thermo Fisher Scientific) with a UPLC BEH Amide column (2.1 mm × 100 mm, 1.7 μm) coupled to Q Exactive HFX mass spectrometer (Orbitrap MS, Thermo). The mobile phase consisted of 25 mmol/L ammonium acetate and 25 ammonia hydroxide in water（pH = 9.75）(A) and acetonitrile (B). The analysis was carried with elution gradient as follows: 0~0.5 min, 95%B; 0.5~7.0 min, 95%~65% B; 7.0~8.0 min, 65%~40% B; 8.0~9.0 min, 40% B; 9.0~9.1 min, 40%~95% B; 9.1~12.0 min, 95% B. The column temperature was 30 ℃. The auto-sampler temperature was 4 ℃, and the injection volume was 3 μL.

The QE HFX mass spectrometer was used for its ability to acquire MS/MS spectra on information-dependent acquisition (IDA) mode in the control of the acquisition software (Xcalibur, Thermo). The ESI source conditions were set as following: sheath gas flow rate as 50 Arb, Aux gas flow rate as 10 Arb, capillary temperature 320 ℃, full MS resolution as 60000, MS/MS resolution as 7500, collision energy as 10/30/60 in NCE mode, spray Voltage as 3.5 kV (positive) or -3.2 kV (negative), respectively. The raw data were converted to the mzXML format using ProteoWizard and processed with an in-house program, which was developed using R and based on XCMS, for peak detection, extraction, alignment, and integration. Then a MS2 database (BiotreeDB) was applied in metabolite annotation. The extraction, identification and quantitation of fecal metabolites was completed by the Biotree biotech Co., Ltd (Shanghai, CN).

The identified metabolites then were quantified and the abundance of them were normalized by log10 transformation. The composition of metabolome was calculated with Partial Least Squares Discriminant Analysis (PLSDA) and the metabolites with day-night difference were selected using fold change (FC) analysis and T-test with absolute value of FC value more than 1 and the adjusted Pvalue (P.adj) less than 0.05. Then, these different metabolites were enriched into some KEGG pathways. All these analyses were performed with a functional R package MetaboAnalystr (v3.0.3).

**Cellular RNA extraction and** **quantitative real-time PCR**

The 100 μM methionine or 10% PBS treated Caco-2 cells were confluenced to 90% after 24 hours, and then were digested with 0.05% trypsin to prepare a cell suspension, followed by centrifugation at 800$\times$g for 5 minutes to remove the trypsin and make cell precipitate. The cellular total RNA was extracted from these Caco-2 cells with an RNA Easy Fast Tissue/Cell Kit (Tiangen, CN), referring to the manufacturer’s instructions. The total RNA solutions were qualified using NanoDrop (Thermo Fisher Scientific, US) and all samples had a 260/280 above 2. Then the part of these RNA solutions was reverse transcribed using a Fastking RT kit (with gDNase) (Tiangen, CN), in which the genomic DNA was removed, and the remaining RNAs were stored at -80 °C. The quantitative real-time PCR (qPCR) was performed and analyzed using an Applied Biosystems 7500 Fast Real-Time PCR System with SYBR green reagent in the KAPA SYBR® FAST kit (KAPA Biosystems, US) and 10 μM primers (see sequences in Table S1). PCR conditions were 3 min at 95 °C followed by 40 cycles of 15 s at 95 °C, 30 s at 56 °C. Relative expression levels were determined using the comparative CT method to normalize target gene mRNA to *GAPDH* (glyceraldehyde-3-phosphate dehydrogenase) gene.

**Nanopore direct RNA sequencing and methylation detection**

The extracted and purified cellular RNA of Caco-2 cells mentioned earlier were utilized to prepare direct RNA library according to the manufacturer’s instructions for direct RNA sequencing (Nanopore Technologies, SQK-RNA002, Oxford, UK). Then, the RNA library was directly sequenced on the nanopore technology PromethION platform (Zhao et al., 2021). The nanopore sequencing data was basecalled using Guppy (v5.0.11+2b6dbff) and then was used to detect m6A and 5mC with Xpore (v2.0) (Pratanwanich et al., 2021) and Tombo (v1.5.1) (Stoiber et al., 2017). The identified m6A modified sites were screened with limited differential modification rates (DMRs) and adjusted Pvalue (DMR > 0.5; p.adj < 0.001). Similarly, the reliable 5mC modified sites were selected with the criteria that the modified sites must be sequenced to a coverage greater than 10x and have a methylation occurrence greater than 0.7. Then, the modified sites were mapped to transcripts and the methylation levels of these transcripts were calculated to investigate the overall cellular m6A and 5mC level across conditions. Then, the important transcripts with modified sites were aligned to gene coordinates and levels of m6A and 5mC modification were calculated. After that, a preliminary fold change analysis was performed (without T-test) to select highly methylated genes in methionine-treated Caco-2 cells (FC value > 1), the rhythm-related genes in which were enriched into the GO (Gene Ontology) terms using clusterProfiler R package (v3.10.1).

**Cellular ROS detection**

Caco-2 cells were cultured in 6-well plates treated 100 μM methionine or 10% PBS until confluent. Then cells were digested with 0.5% trypsin (GIBCO, 25200-056) to prepare cell suspensions, which were latter centrifuged and washed with PBS to get purified cells. Next, the cells were resuspended in fresh medium containing 5μM SYTOX® Red Dead Cell Stain and incubate for 30 minutes at 37°C, protected from light, referring to the manufacture’s instruction of a CellROX® Flow Cytometry Assay Kit (Thermo Fisher Scientific, US). After that, cells were washed and once and resuspended with PBS. Immediate analysis was performed by flow cytometry, using 488-nm excitation and collecting fluorescence emission with a 530/30 BP filter as instruction.

**Western blot**

Cells were prepared as mentioned above and protein was extracted using RIPA buffer, lysis buffer with the protease inhibitor phenylmethane sulfonyl fluoride (all from Beyotime, CN). The concentrations of extracted protein then were measured using the BCA protein assay kit (Beyotime, CN). Equivalent amounts of protein (20 μg) were incubated 10 minutes at 100 ℃ for denaturation and then separated on 10% SDS-polyacrylamide gel electrophoresis gels (Biorad, US) for 27 minutes at 250 V. Next, the separated proteins were transferred on to 0.45 μm polyvinyl difluoride membranes (Billerica, US) according to instructions. Then the polyvinyl difluoride membranes were incubated in blocking buffer, which is made by dissolving milk powder Tris-buffered saline with Tween-20 (TBST) thoroughly, at room temperature for 1 h. The membranes were incubated with primary antibodies, including anti-GAPDH antibody (ab8245; Abcam, UK), anti-HSP90 antibody (4877S; CST, US), anti-SOD1 antibody (ab13498; Abcam, UK), anti-Catalase antibody (ab16731; Abcam, UK), and anti-SOD2 antibody (ab13533; Abcam, UK), at 4°C overnight. After washed three times with TBST, the membranes were incubated with a secondary antibody, goat anti-rabbit (Santa Cruz Biotechnology, US) for 1 h at room temperature. Then, the membranes were washed three times with TBST for 5 minutes each and proteins were detected using ECL reagent and quantified using the ChemiDoc MP System (Biorad, US).

**Sleep deprivation in mice**

To investigate the impacts of methionine during sleep deprivation (SD) *in vivo*, dietary methionine manipulation in mice were performed in advance. The 30 male mice (7-8 weeks old) were parted into 4 groups (n > 6 per group), which are dieting on chow containing 0%, 0.86% and 1.36% of methionine for 5 days (Ji et al., 2021). Among these 4 groups, two were fed with 0.86% methionine diet as dietary controls. The Met-free chaw (LAA-0Met) and chaw containing 0.86% (D191027) were purchased from Dyets, Inc. (Bethlehem, PA). The 1.36% methionine-rich diet was composed by 0.86% methionine chow and additional 0.5% Methionine in the water. The continuous SD was performed in three restriction chambers (8500-K1; Pinnacle Technology, US), each having a rotating bar keeping constant but gentle motion (speed at 6 all time) to limit mice sleep. All mice except one of the two groups fed with 0.86% methionine diet were subjected to SD for 3 days totally. During SD, mice had full access to the same food and water as the previous 5 days.

**Serum corticosterone measuring**

To assess how the SD and methionine impact the stress levels of mice, 1 ml of trunk blood (eyeball enucleation) was collected before dissection, and then was centrifuged to get serum. Corticosterone levels in SD and non-SD murine serum were measured by Corticosterone Parameter Assay Kit (R&D Systems, UK) according to the instruction.

**ROS detection in murine tissues**

Control and sleep-deprived mice were sacrificed at 3 days after the onset of SD. Small intestines were collected and washed in pre-cooled PBS. A 5-centimeter small intestine segment of each mouse near the colon then was rolled into “swiss roll” as previously reported (Moolenbeek and Ruitenberg, 1981). The rolls were immediately embedded in O.C.T (Tissue-Tek, US) and then frozen in liquid nitrogen. Next, with a Leica cryostat (CM3050 S; Leica Biosystems, US), the tissues were sectioned into 6 μm sections, which were mounted on microscope glass slides (VWR, US) immediately and incubated with 10 μM DHE (Sigma-Aldrich) for 30 minutes at 37°C. Then, these sections were washed with PBS three times for 5 minutes each and Fluoromount-G (SouthernBiotech, US) was utilized to stain sections and fix coverslips. After that, sections were imaged with Vectra Polaris (Akoya Biosciences, US). H_2_O_2_ levels of intestines, livers and brains of SD and non-SD mice were determined using a Hydrogen peroxide assay kit (Solarbio, CN) and its absorbance was measured at 415 nm.

**RNA sequencing and data analysis**

Total RNA was prepared from livers, small intestines and blood cells mice in SD experiment with an RNA Easy Fast Tissue/Cell Kit (Tiangen, CN), according to the manufacturer’s instructions. RNA integrity was assessed using the Fragment Analyzer 5400 (Agilent Technologies, CA, USA). Sequencing libraries were prepared by using NEBNext® UltraTM RNA Library Prep Kit (NEB, USA) following manufacturer’s recommendations Kit and sequenced on an Illumina Novaseq 6000 platform and 150 bp paired-end reads were generated. The RNA sequencing was completed by Novogene Co., Ltd (Beijing, CN). The sequencing reads with low quality were filtered out using fastp (v0.23.1) (Chen et al., 2018) and then the qualified reads were aligned to reference genome assembly mm10 using Hisat2 (v2.2.1) (Kim et al., 2019) with default parameters. Aligned reads then were mapped to genomic features and quantified using StringTie (v2.1.7) (Pertea et al., 2015). Next, counts were calculated and utilized to quantify the relative abundance of genes. Differential expression analysis was performed using DESeq2 (v1.22.2) package and the differentially expressed genes were identified with the criteria of 2-fold change value in expression and P.adj less than 0.05.

**Statistical Analysis**

All experimental results are expressed as mean ± SEM and data were analyzed by unpaired two-tailed student's t test (Sun et al., 2020) and the relative abondance of bacterial species was compared using unpaired wilcoxon test (Qin et al., 2022). *p-value < 0.05, **p-value < 0.01, ***p-value < 0.005; ****p-value < 0.001.

**Supplemental Reference**

Bolger, A.M., Lohse, M., and Usadel, B. (2014). Trimmomatic: a flexible trimmer for Illumina sequence data. Bioinformatics 30, 2114-2120.

Chen, S., Zhou, Y., Chen, Y., and Gu, J. (2018). fastp: an ultra-fast all-in-one FASTQ preprocessor. Bioinformatics 34, i884-i890.

Franzosa, E.A., McIver, L.J., Rahnavard, G., Thompson, L.R., Schirmer, M., Weingart, G., Lipson, K.S., Knight, R., Caporaso, J.G., Segata, N.*, et al.* (2018). Species-level functional profiling of metagenomes and metatranscriptomes. Nature Methods 15, 962-968.

Ji, M., Xu, X., Xu, Q., Xu, X., Azcarate-Peril, M.A., Wu, X., Liu, J., Locasale, J.W., Li, J.-L., Shats, I.*, et al.* (2021). Dietary methionine restriction impairs anti-tumor immunity through gut microbiota. bioRxiv, 2021.2008.2027.457955.

Kim, D., Paggi, J.M., Park, C., Bennett, C., and Salzberg, S.L. (2019). Graph-based genome alignment and genotyping with HISAT2 and HISAT-genotype. Nat Biotechnol 37, 907-915.

Moolenbeek, C., and Ruitenberg, E.J. (1981). The "Swiss roll": a simple technique for histological studies of the rodent intestine. Lab Anim 15, 57-59.

Pertea, M., Pertea, G.M., Antonescu, C.M., Chang, T.C., Mendell, J.T., and Salzberg, S.L. (2015). StringTie enables improved reconstruction of a transcriptome from RNA-seq reads. Nat Biotechnol 33, 290-295.

Pratanwanich, P.N., Yao, F., Chen, Y., Koh, C.W.Q., Wan, Y.K., Hendra, C., Poon, P., Goh, Y.T., Yap, P.M.L., Chooi, J.Y.*, et al.* (2021). Identification of differential RNA modifications from nanopore direct RNA sequencing with xPore. Nature Biotechnology 39, 1394-1402.

Qin, Y., Havulinna, A.S., Liu, Y., Jousilahti, P., Ritchie, S.C., Tokolyi, A., Sanders, J.G., Valsta, L., Brożyńska, M., Zhu, Q.*, et al.* (2022). Combined effects of host genetics and diet on human gut microbiota and incident disease in a single population cohort. Nat Genet 54, 134-142.

Segata, N., Izard, J., Waldron, L., Gevers, D., Miropolsky, L., Garrett, W.S., and Huttenhower, C. (2011). Metagenomic biomarker discovery and explanation. Genome Biol 12, R60.

Segata, N., Waldron, L., Ballarini, A., Narasimhan, V., Jousson, O., and Huttenhower, C. (2012). Metagenomic microbial community profiling using unique clade-specific marker genes. Nat Methods 9, 811-814.

Stoiber, M., Quick, J., Egan, R., Eun Lee, J., Celniker, S., Neely, R.K., Loman, N., Pennacchio, L.A., and Brown, J. (2017). &lt;em&gt;De novo&lt;/em&gt; Identification of DNA Modifications Enabled by Genome-Guided Nanopore Signal Processing. bioRxiv, 094672.

Sun, N., Jiang, L., Ye, M., Wang, Y., Wang, G., Wan, X., Zhao, Y., Wen, X., Liang, L., Ma, S.*, et al.* (2020). TRIM35 mediates protection against influenza infection by activating TRAF3 and degrading viral PB2. Protein Cell 11, 894-914.

Zhao, N., Cao, J., Xu, J., Liu, B., Liu, B., Chen, D., Xia, B., Chen, L., Zhang, W., Zhang, Y.*, et al.* (2021). Targeting RNA with Next- and Third-Generation Sequencing Improves Pathogen Identification in Clinical Samples. Advanced Science n/a, 2102593.

**Supplemental Figure**


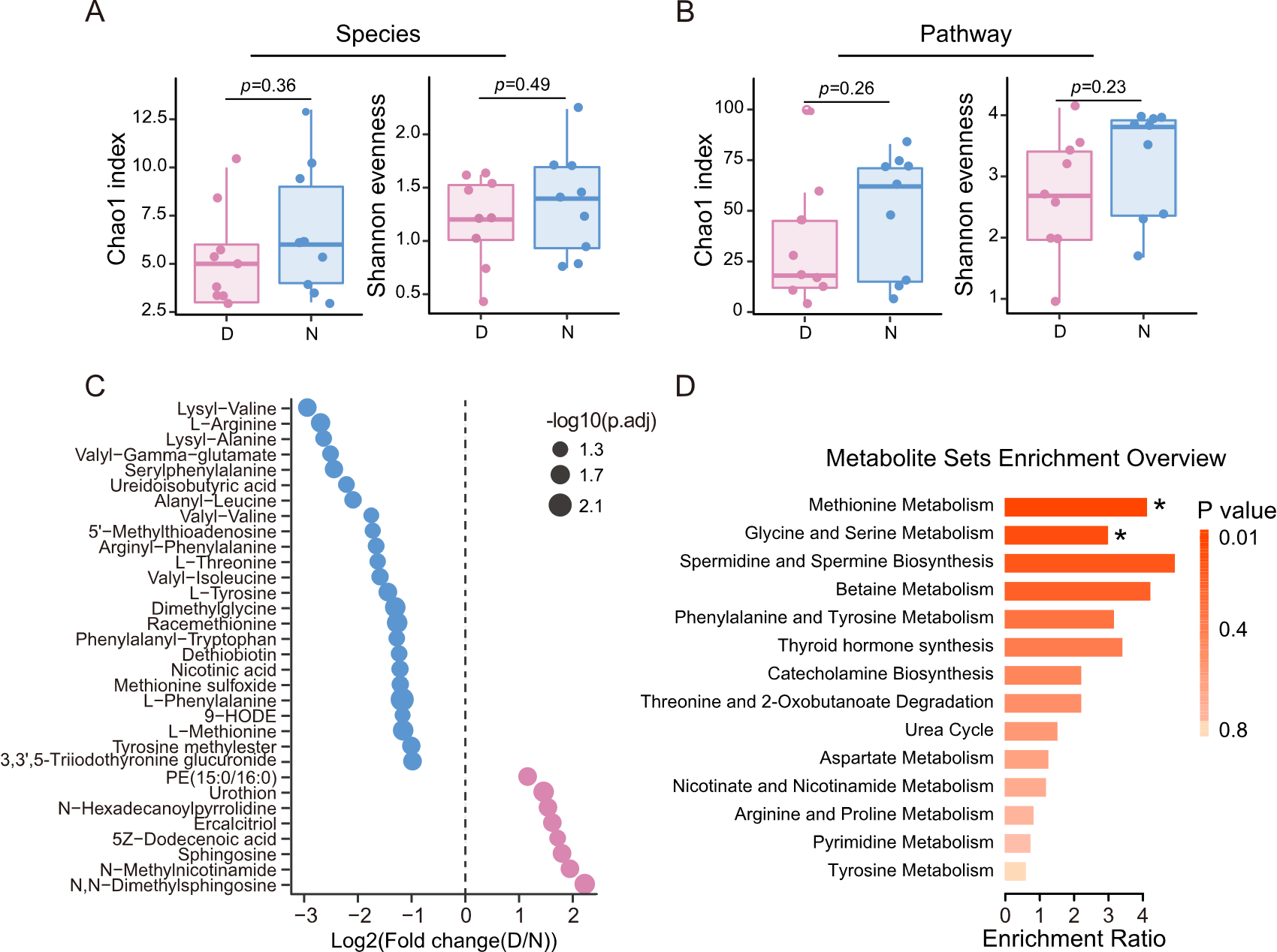


**Fig. S1 Day-night differences were observed in composition, function and metabolites of gut microbiome.** (A) Alpha-diversities (Chao1 index and Shannon evenness) of species composition during the daytime (D) or the night time (N). Statistical significance was calculated using Wilcoxon test. (B) Alpha-diversities of metabolic pathways during the daytime (D) or the night time (N). Statistical significance was calculated using Wilcoxon test. (C) The details of twenty-four metabolites accumulated at night (blue dots) and eight metabolites enriched at day (pink dots). The size of dots indicates the significance of metabolites. (D) The KEGG pathways enriched by the metabolites with day-night difference.


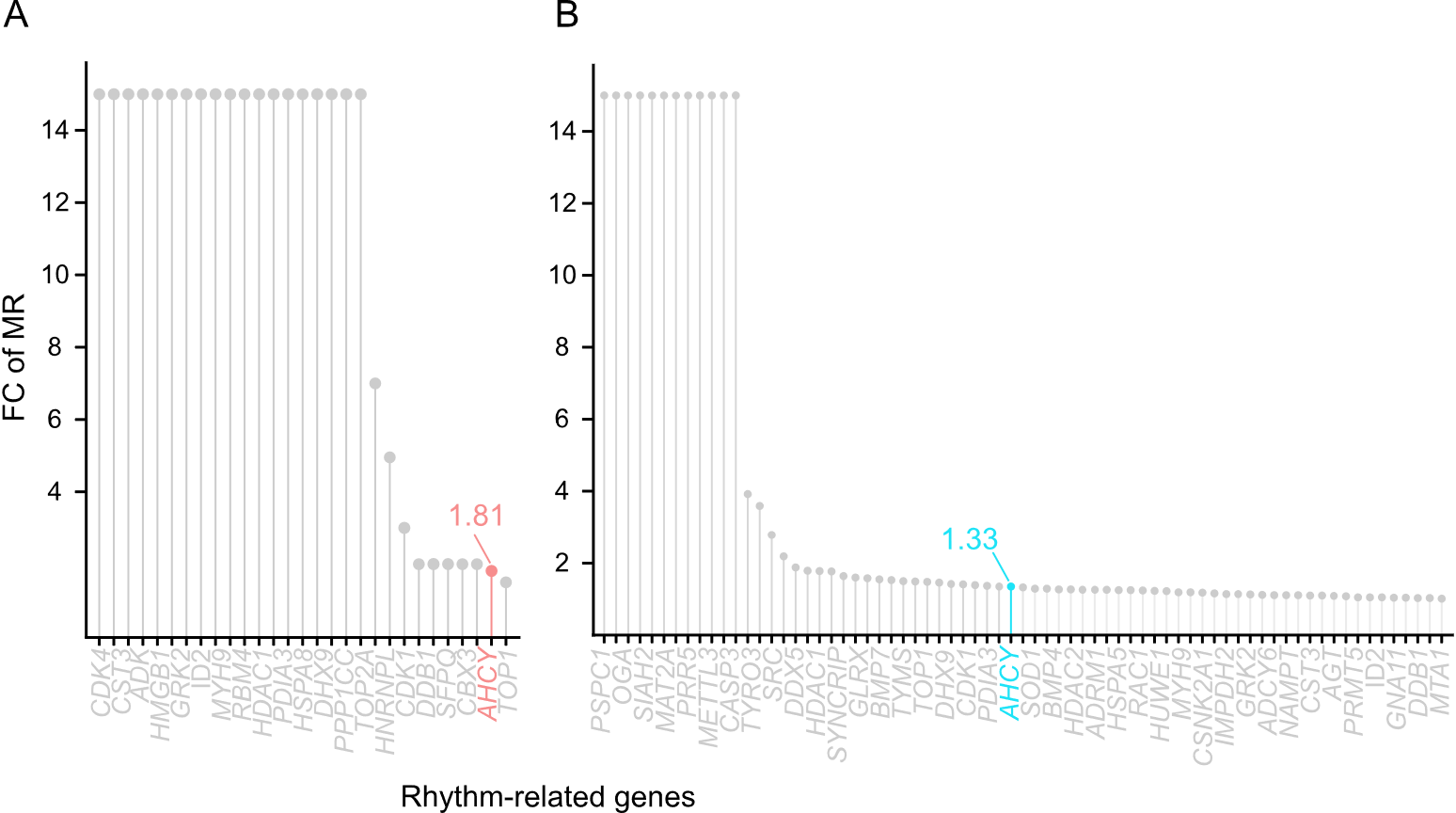


**Fig. S2 The increased methylation levels of** **rhythm-related genes.** (A) The increased m6A levels of rhythm-related genes in methionine-treated Caco-2 cells, with FC (Foldchange) of MR > 1. (B) The increased 5mC levels of rhythm-related genes in methionine-treated Caco-2 cells, with FC of MR > 1.


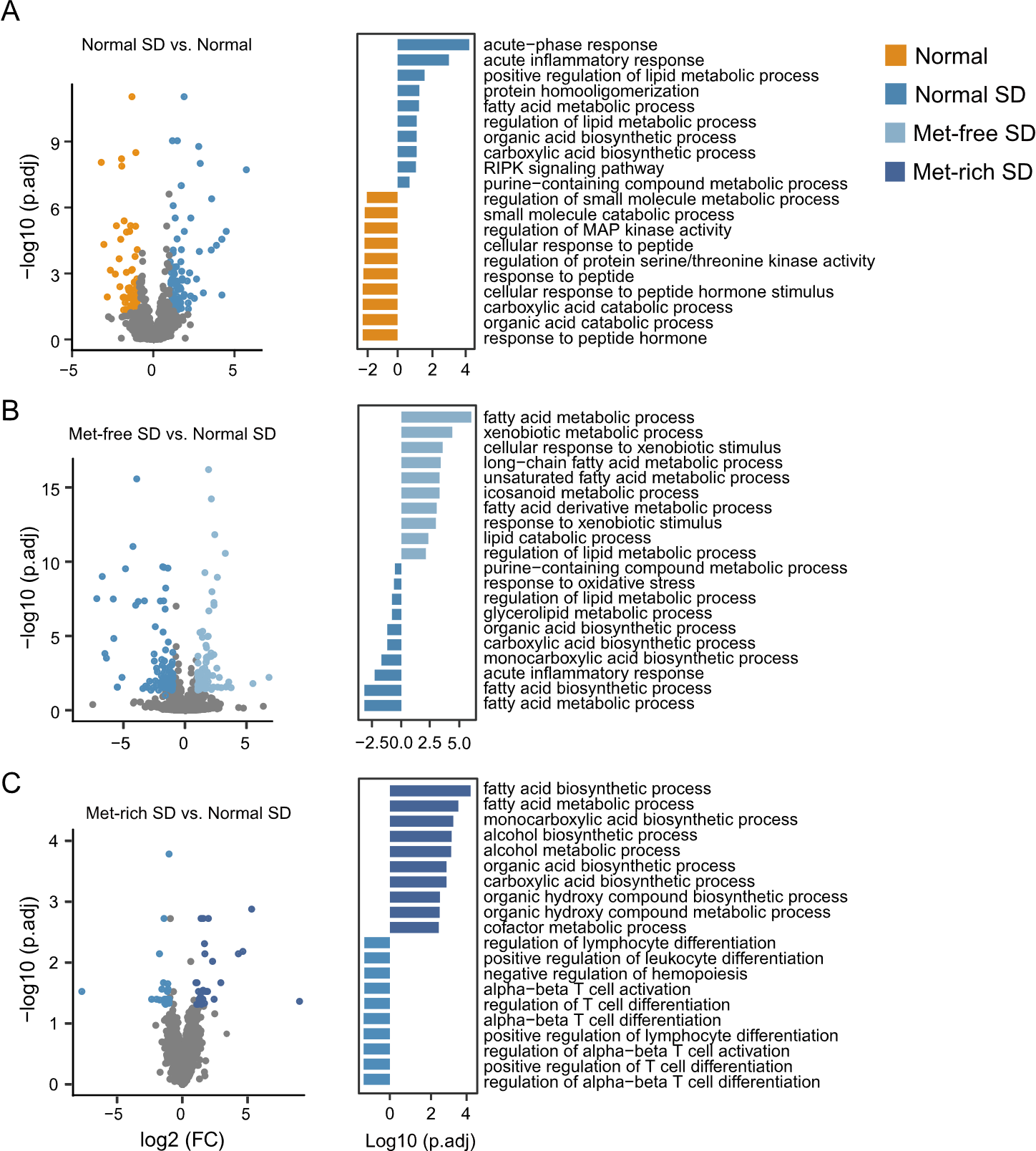


**Fig. S3 Differentially expressed genes and the corresponding biological functions in murine livers.** (A) volcano plot (left panel) showing genes with different expression levels comparing SD and non-SD mice with normal dietary methionine level (Normal SD vs. Normal). The bar plot (right panel) indicating most enriched biological functions (TOP 10) in GO enrichment analysis of the differentially expressed genes comparing the Normal SD group and Normal group. (B-C) volcano plot (left panel) showing genes with different expression levels comparing SD mice with normal dietary methionine and methionine deprived (B, Met-free SD vs. Normal SD) or methionine supplemented (C, Met-rich SD vs. Normal SD). The bar plot (right panel) indicating most enriched biological functions (TOP 10) in GO enrichment analysis of the differentially expressed genes comparing the Met-free SD group and Normal SD (B) or comparing the Met-rich SD group and Normal SD (C). The colored dots and bars represent differentially expressed genes with log2 (FC) > 1, p.adj < 0.05 and the GO enrichment analysis significance declared at p < 0.05 and p.adj < 0.20.


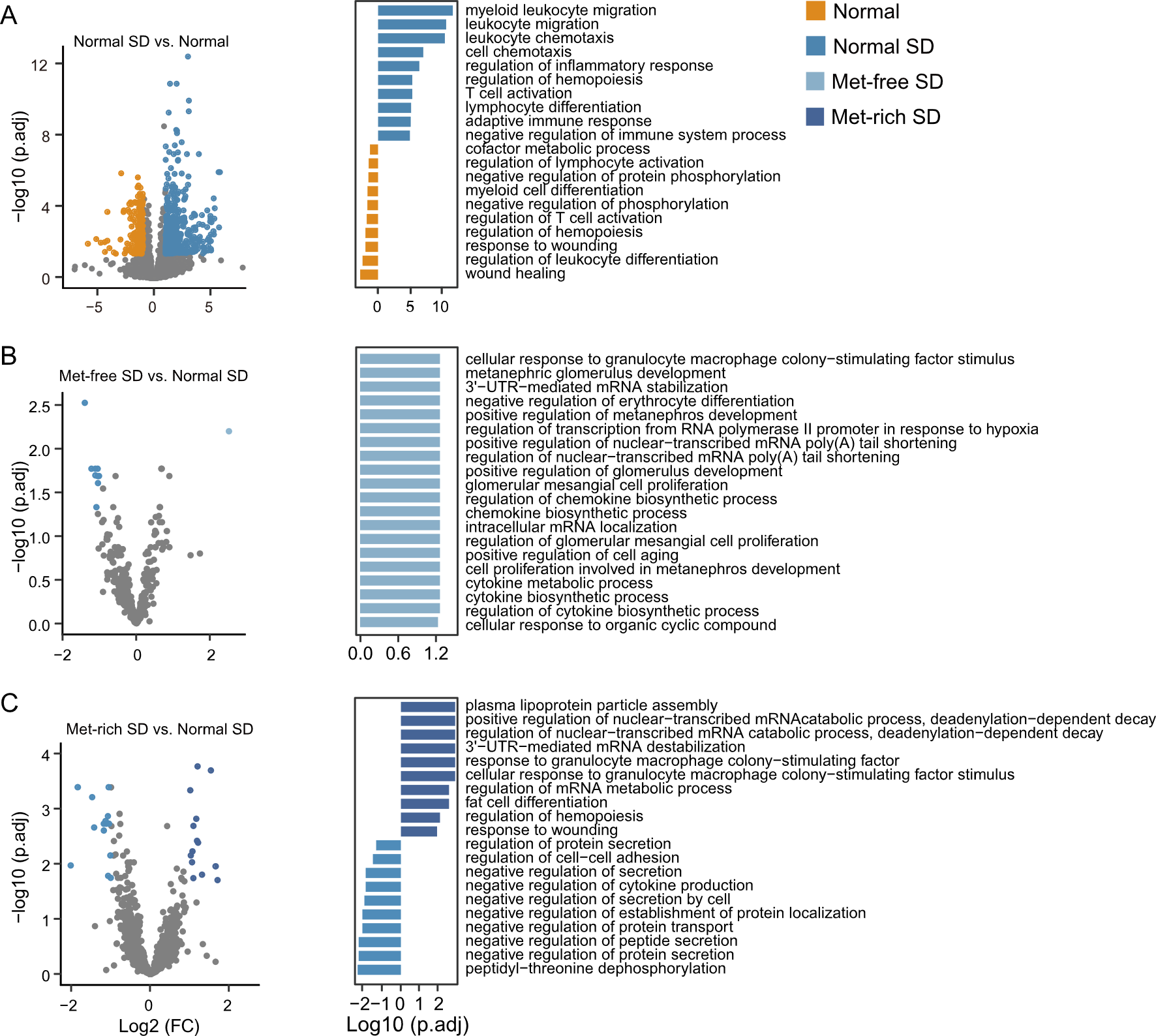


**Fig. S4 Differentially expressed genes and the corresponding biological functions in murine blood cells.** (A) volcano plot (left panel) showing genes with different expression levels comparing SD and non-SD mice with normal dietary methionine level (Normal SD vs. Normal). The bar plot (right panel) indicating most enriched biological functions (TOP 10) in GO enrichment analysis of the differentially expressed genes comparing the Normal SD group and Normal group. (B-C) volcano plot (left panel) showing genes with different expression levels comparing SD mice with normal dietary methionine and methionine deprived (B, Met-free SD vs. Normal SD) or methionine supplemented (C, Met-rich SD vs. Normal SD). The bar plot (right panel) indicating most enriched biological functions (TOP 10) in GO enrichment analysis of the differentially expressed genes comparing the Met-free SD group and Normal SD (B) or comparing the Met-rich SD group and Normal SD (C). The colored dots and bars represent differentially expressed genes with log2 (FC) > 1, p.adj < 0.05 and the GO enrichment analysis significance declared at p < 0.05 and p.adj < 0.20.


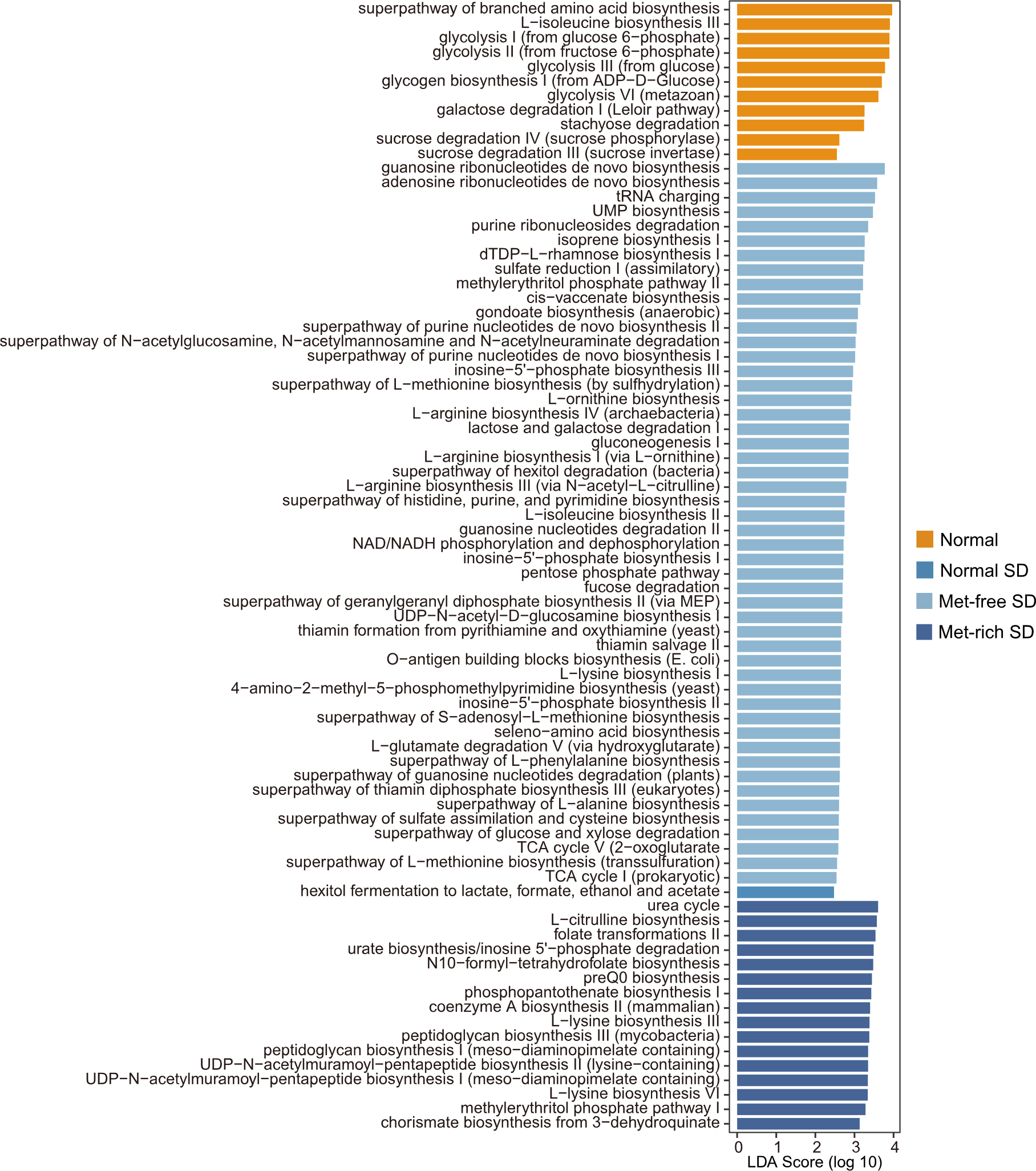


**Fig. S5 Differentially activated metabolic pathways of mouse gut microbiota across groups.** Linear discriminant analysis (LDA) effect size (LEfSe) results represented significantly differential pathways in different groups, with LDA score (log 10) > 2 and p.adj < 0.05.

**Tab. S1 Primer sequences in quantitative real-time PCR analyses**

| **Gene** | **Forward Primer (5′ - 3′)** | **Reverse Primer (5′ - 3′)** |
| --- | --- | --- |
| *BMAL1* | GCTCAGGAGAACCCAGGTTATC | GCATCTGCTTCCAAGAGGCTCA |
| *CLOCK* | CAGGCAGCATTTACCAGCTCATG | GTAGCTTGAGACATCACTGGCTG |
| *PER2* | CGACGTGGCAGAATGTGTT | GATTCAAGGGGGATCCATTT |
| *CRY1* | GCAGTTGCTTGCTTCCTGACAC | GACAGCCACATCCAACTTCCAG |
| *NR1D2* | GGCATGGTTCTACTGTGTAA | AGTTTTCATTCTTCAGGCAC |
| *AHCY* | ATCCTCAAGGTGCCTGCCATCA | CGGCAATCATCACATCTGTGGC |
| *GAPDH* | AGCCACATCGCTCAGACAC | GCCCAATACGACCAAATCC |
